# Development of KoRV-pseudotyped lentiviral vectors for efficient gene transfer into freshly isolated immune cells

**DOI:** 10.1038/s41434-024-00454-0

**Published:** 2024-04-29

**Authors:** Alexander Renner, Anika Stahringer, Katharina Eva Ruppel, Stephan Fricke, Ulrike Koehl, Dominik Schmiedel

**Affiliations:** 1 Fraunhofer Institute for Cell Therapy and Immunology (IZI), Department for Cell and Gene Therapy Development, Leipzig, Germany; 2 Fraunhofer Cluster of Excellence for Immune-Mediated Diseases, CIMD, Leipzig, Deutschland; 3 Institute for Clinical Immunology, University of Leipzig, Leipzig, Germany

**Keywords:** Genetic transduction, Tumour immunology, Immunotherapy, Genetic vectors, Innate immune cells

## Abstract

Allogeneic cell therapies, such as those involving macrophages or Natural Killer (NK) cells, are of increasing interest for cancer immunotherapy. However, the current techniques for genetically modifying these cell types using lenti- or gamma-retroviral vectors present challenges, such as required cell pre-activation and inefficiency in transduction, which hinder the assessment of preclinical efficacy and clinical translation. In our study, we describe a novel lentiviral pseudotype based on the Koala Retrovirus (KoRV) envelope protein, which we identified based on homology to existing pseudotypes used in cell therapy. Unlike other pseudotyped viral vectors, this KoRV-based envelope demonstrates remarkable efficiency in transducing freshly isolated primary human NK cells directly from blood, as well as freshly obtained monocytes, which were differentiated to M1 macrophages as well as B cells from multiple donors, achieving up to 80% reporter gene expression within three days post-transduction. Importantly, KoRV-based transduction does not compromise the expression of crucial immune cell receptors, nor does it impair immune cell functionality, including NK cell viability, proliferation, cytotoxicity as well as phagocytosis of differentiated macrophages. Preserving immune cell functionality is pivotal for the success of cell-based therapeutics in treating various malignancies. By achieving high transduction rates of freshly isolated immune cells before expansion, our approach enables a streamlined and cost-effective automated production of off-the-shelf cell therapeutics, requiring fewer viral particles and less manufacturing steps. This breakthrough holds the potential to significantly reduce the time and resources required for producing e.g. NK cell therapeutics, expediting their availability to patients in need.

## Introduction

Cell therapy has emerged as a groundbreaking approach in modern medicine, holding the promise to revolutionize the treatment of various diseases [1]. However, although Chimeric Antigen Receptor (CAR-) T cell therapy achieved very good results in recent years for several malignancies of hematopoietic origin [2, 3] and have been approved for treating various B cell malignancies and multiple myeloma patients, their effectiveness in treating solid tumors is still limited [4, 5]. Therefore, additional cell types like Natural Killer (NK) cells and macrophages are being evaluated for their potential in cancer therapy, with the additional advantage of being applicable as an allogeneic cellular therapy. Whereas T cells have to be heavily engineered to be applied in allogeneic settings without causing graft-vs-host-disease (GvHD) due to Human Leukocyte Antigen (HLA) mismatch between donor and recipient, NK cells and macrophages are considered safe to be transferred in allogeneic settings [6, 7].

Natural Killer (NK) cells have opened up the field of allogeneic cellular therapies, and several (CAR-) NK cell products have been tested in (pre-)clinical studies [8–10]. One of the key advantages of NK cells in tumor therapy is their innate cytotoxicity. They rapidly recognize and eliminate malignant cells without prior sensitization and predominantly recognize transformed cells based on stress-induced ligands through an array of invariant receptors [11]. In contrast to T cells, NK cell activation is predominantly inhibited by surface HLA class I molecules [12]. This characteristic makes NK cells particularly valuable in cases where downregulation of HLA expression by cancer cells limits T cell recognition and mediates immune evasion. Additionally, in the context of CAR NK cells, antigen loss or heterogeneity observed for patients both in CD19- and BCMA-directed CAR-T cell therapies, may be less critical due to the NK cells’ ability to target a broader spectrum of tumor ligands [13, 14]. A clinical phase I trial using allogeneic CD19-directed NK cells showed potent anti-tumor effects without the occurrence of severe side effects [15].

Another promising cell type for the development of allogeneic cell therapies are macrophages. The innate functions of macrophages involve the removal of apoptotic cells in tissues and the protection from foreign substances such as bacteria through the process of phagocytosis in the first line of defense [16]. Macrophages emerged as a cell therapy to rebuild macrophage populations in patients with inborn immune defects [17, 18]. However, with the continuous struggle of CAR T cells to efficiently tackle solid tumors, the potential of genetically engineered macrophages in immunotherapies to destroy tumor cells is currently under intense investigation [19, 20]. The limitation of T or NK cell therapies to battle solid tumors might be overcome by macrophages which, in comparison to T and NK cells, efficiently infiltrate and populate the tumor microenvironment (TME) and therefore might represent a more efficient therapy option especially for solid tumors [21]. Most CAR macrophage studies are still preclinical [21–24]. One study using CAR macrophages directed against the HER2 antigen showed target-specific phagocytosis and tumor clearance in vitro, while overall survival in a mouse model could be prolonged [24].

B cells are also coming into focus for cell therapy, mainly because of the invaluable therapeutic potential of monoclonal antibodies [25]. Here, their potential for long-term secretion of antibodies and memory cell formation sets out to be very promising for the treatment of autoimmune diseases or cancer as well as the prevention or treatment of infectious diseases [26–28]. In the tumor microenvironment, B cells also have the capacity to stimulate other immune cells, through secretion of cytokines, promotion of Type 1 T helper (Th1) cells and activation of cytotoxic T cells [29, 30].

However, this shift from autologous therapies mainly using T cells to allogeneic approaches involving NK cells, B cells and macrophages raises new challenges. One central point to the success of cell therapies is the efficient and targeted delivery of therapeutic transgenes into immune cells. Historically, NK cells have always been hard to modify by viral gene transfer and major progress has only been made a few years back. Gene transfer either enables effector cells to perform targeted activities, like the recognition of specific antigens to kill tumor cells as in CAR therapy, or enhances their natural effector functions. Low efficiency in gene transfer and expression - especially at early points of cell cultivation - has so far been slowing the field as well as causing long-lasting and expensive production processes [31, 32].

The most common method for gene delivery to immune cells is retro- or lentiviral transduction in which replication-incompetent viruses are used to transfer genes into cells and ensure integration into the genome [33]. Pseudotyped lentiviral vectors equipped with a variety of envelope glycoproteins have gained significant importance due to their ability to mediate robust gene transfer and long-term transgene expression in diverse cell types [34]. Especially Vesicular Stomatitis Virus (VSVG) envelope glycoprotein-based pseudotyped lentiviral vector (LV) has emerged for the genetic modification of T cells [35], with Feline Endogenous Retrovirus envelope protein (RD114-TR)-pseudotyped viral particles and Gibbon Ape Leukemia Virus (GALV) being suitable alternatives [36]. In NK cell research, RD114-TR-pseudotyped viral particles and Baboon Endogenous Virus (BaEV)-based LVs have been demonstrated to achieve efficient transduction rates in cytokine-stimulated NK cells [37–39]. Interestingly, well-known lentiviral vectors have encountered hurdles in achieving high transduction efficiencies on freshly isolated cells and require pre-stimulation and cultivation of cells prior to transduction. In consequence, current transduction protocols are time-consuming, contain several process steps, and are therefore costly. Novel and efficient approaches to enhance transgene delivery and shorten manufacturing protocols for cell therapy products are under intense investigation [37]. In this study, we show the exceptional potential in revolutionizing the manufacturing processes to generate Advanced Medicinal Therapeutic Products (ATMPs) from freshly isolated immune cells, by developing lentiviral vectors based on two variants of the envelope glycoprotein of the exogenous retrovirus Koala Retrovirus (KoRV), KoRVA and KoRVB [40].

## Methods

### Primary cells and culture conditions

Buffy coats from healthy donors were obtained from the Institute for Transfusion Medicine of the university clinic in Leipzig, Germany (ethical vote number 327/22-ek). Cells were then isolated by standard density-gradient centrifugation using Ficoll-Paque (VWR, Germany, Catalog. Nr. 17-1440-03) using the RosetteSep™ Human NK Cell Enrichment Cocktail (Stemcell Technologies, Canada) for NK cells, the RosetteSep™ Human B Cell Enrichment Cocktail for B cells and the RosetteSep™ Human Monocyte Enrichment Cocktail for monocytes, respectively. NK cells were cultured at 1 × 10^6^ cells/mL in NK MACS medium (Miltenyi Biotec, Germany) with 5% human AB serum (Sigma Aldrich, USA, Catalog Nr. H4522-100ML), 500 U/mL IL-2 and 140 U/mL IL-15 (Peprotech, USA). B cells were cultured at 1 × 10^6^ cells/mL in RPMI 1640 medium (Thermo Fisher Scientific, Catalog Nr. 11875093, USA) with 5% fetal calf serum (FCS, Bio&Sell, Germany), 10 ng/mL human CD40L (AdipoGen Life Sciences, Catalog Nr. AG-40B-0010-C010, USA) and 1 ng/mL human IL-4 (Miltenyi, Catalog Nr. 130-095-373, Germany). Monocytes were cultured at 1 × 10^6^ cells/mL in RPMI 1640 medium with 5% FCS and 20 ng/mL recombinant human GM-CSF (Leukine®, sargramostim).

### Plasmids and viral production

Lentiviral vector (LV) containing supernatants were generated via transient transfection of HEK293T cells with the use of TransIT transfection reagent (Mirus Bio, USA) according to the manufacturer’s instructions. Briefly, 1.5 × 10^5^ HEK293T cells were seeded in one well of a 6-well plate one day prior to transfection. For LV production, on the day of transfection, 1 µg of the packaging plasmids in a 4:1:1 mixture (pMDLg/pRRE-gagpol:pRSV-Rev:Env – meaning 0.667 µg pMDLg/pRRE-gagpol and 0.167 µg of pRSV-Rev and Env each) and 1 µg of the transgene plasmid were mixed in 200 µL Dulbecco’s Modified Eagle Medium (DMEM, Thermo Fisher Scientific, USA) and 6 µL of TransIT transfection reagent were added for production of LVs. Third generation packaging plasmids pMDLg/pRRE-gagpol and pRSV-Rev (Addgene #12251 and #12253, kindly provided by Didier Trono) were used alongside the transfer vector hEF1α-H2B-mVenus-IRES-mCherry PGK-Puromycin (Addgene #60141, kindly provided by Anna-Katerina Hadjantonakis) and an envelope protein for pseudotyping. Gibbon Ape Leukemia Virus (GALV) (was kindly provided by Dr. Jan Münch, University Ulm) [36, 41], KoRVA (GenBank: BAN63359.1), KoRVB (GenBank: AGO86848.1), RD114-TR [39] and BaEV [42] envelope glycoproteins were used. Sixteen hours after transfection, cell culture medium was exchanged and two days after medium exchange, the virus containing cell culture supernatant was collected and filtered using a 0.45 µm syringe filter. LV containing supernatants were stored at −80 °C. For normalization of viral titers to KoRVA LV, LV supernatants were diluted using DMEM according to gene copy numbers determined in qRT-PCR.

### Reverse transcription qPCR

Gene copy number of LV was determined by Lenti-X™ qRT-PCR Titration Kit (Takara Bio Inc, Japan). Briefly, RNA was isolated from virions via NucleoSpin® RNA Virus kit (Machery-Nagel, Germany). Then, purified RNA was used for reverse transcription and qPCR on a LightCycler® 480 System (Roche, Germany). The thermal cycling conditions were chosen according to manufacturer’s instructions and the experiment was carried out in duplicates. Gene copy numbers were determined through a standard curve resulting from RNA samples of known concentrations included within the kit.

### Lentiviral transduction

Immediately after isolation, primary immune cells were transduced with lentiviral particles. NK cells (1.25 × 10^5^) were seeded per well in a 48-well plate. For PBMC, B cells and monocytes, 2.5 × 10^5^ cells were seeded per well in a 48-well plate. 250 µL of diluted or undiluted lentiviral supernatant were mixed with the transduction enhancer Vectofusin-1 (Miltenyi Biotec, Germany) to a final concentration of 10 µg/mL and then incubated at room temperature for 10 min prior to adding to the cells. After spinfection at 37 °C and 400 × *g* for 1 h, 250 µL of fresh complete culture medium was added to the transduced cells and they were cultured in an incubator under standard conditions. Transduction efficiency was assessed via mVenus reporter 3–4 days after transduction using flow cytometry (MACSQuant10, Miltenyi Biotec, Germany).

### Flow cytometric analysis

Flow cytometric analysis was carried out using either a BD Canto, MACSQuant X Analyzer or MACSQuant 10 Analyzer. All NK cell samples were stained with an anti-CD56-APC Vio770 antibody (Miltenyi Biotec, Germany). Transgene expression was then determined on CD56-APC+ cells. For receptor expression, CD69-VioGreen, NKp30-APC, NKp44-PE, NKp46-PE-Vio770, NKG2A-APC, NKG2D-PE, CD16-PerCP-Vio700, CD57-PE-Vio770, KIR2D-PE and NKG2C-APC (Miltenyi Biotec, Germany) were used to stain NK cells. B cell samples were stained with CD19-PE-Vio770 to gate for B cells and then a CD69-VioGreen and CD80-APC antibody (Miltenyi Biotec, Germany) were used for surface receptor characterization. GM-CSF-differentiated monocyte samples were first incubated with Fc-receptor blocking solution (Miltenyi Biotec, Germany) for 30 min on ice and then stained with CD14-APC (Miltenyi Biotec, Germany) and surface expression of receptors was analyzed via staining using CD16-PerCP-Vio700, CD80-APC (Miltenyi Biotec, Germany), HLA-DR-APC and CD86-BV510 (Biolegend, USA).

### Fluorescence microscopy

Microscopic analysis was performed using a Nikon Eclipse Ti-E microscope. Briefly, HEK293T cells were cultured in 6-well plates and transfected as described above. After 3 days of incubation the mVenus expression signal was detected by using a blue laser and a 40 x magnification.

### Calcein assay

Killing of K562 cells was evaluated by fluorescence-based Calcein cytotoxicity assay [43]. Briefly, target cells were labeled with 1 µM Calcein AM (Thermo Fisher Scientific, C1430, USA) according to manufacturer’s instructions and 1 × 10^4^ target cells were seeded into a 96-well U bottom plate. Medium as a control or NK cells in a 1:1 effector to target ratio were added. For maximum Calcein release, K562 cells were treated with 1% Triton X-100. After 2 h of co-incubation, 100 µL of cell culture supernatant were transferred to a black 96-well F bottom plate and Calcein release was detected using a microplate reader (excitation: 494 nm; emission: 517 nm).

### Cell proliferation assay

Proliferation rates of NK cells were determined by using the flow cytometry-based CellTrace Violet Proliferation Kit (Thermo Fisher, Catalog Nr. C34571, USA). Briefly, NK cells were stained with the reagent at a concentration of 1 µM in PBS for 30 min at 37 °C. Subsequently, the reaction was stopped by adding five times the volume of serum-containing cell culture medium and incubation for 10 min. After a medium exchange, CellTrace Violet stained cells were activated in order to induce cell proliferation and cells were cultivated under standard cell culture conditions. The fluorescence signal was evaluated four days after the transduction via flow cytometry. To assess irregularities in proliferation, we assessed the number of dividing cells as well as the amount of cells within each single division. For this purpose, data was analyzed via FlowJo software using the Proliferation tool which counts the peaks created by each cell division and the connected bisection of CellTrace signal in the resulting daughter cells.

### Phagocytosis assay

PBMC-derived macrophages were detached from wells after four days of culturing by placing the cell culture plates on ice for 30 min. Cell culture supernatant was aspirated and cells were washed once using PBS. Then, 500 µL of PBS with 1 mM EDTA were added and left on ice for five more minutes. After the incubation, cells were detached by pipetting.

pHrodo™ BioParticles™ Conjugates in Deep Red (ThermoFisher Scientific, USA) were used for assessment of phagocytosis ability of macrophages after lentiviral transduction. For this purpose, 1.5 × 10^5^ macrophages were incubated with 10 µL of pHrodo™ BioParticles™ Conjugates suspension either on ice to analyze background phagocytosis and possible adhesion of E. coli particles to macrophages or at 37 °C in an incubator under standard cell culture conditions to assess phagocytosis. After incubation for one hour, macrophages were washed once using PBS and then fluorescence in the macrophages was assessed by flow cytometric evaluation.

### Statistical analysis

Statistical analysis was performed using GraphPad Prism 7 software. For comparison of two groups either multiple t-tests with Holm-Sidak correction or Mann–Whitney tests were utilized. Two-way ANOVA was calculated for comparison of three or more groups with Tukey’s multiple comparisons.

### Sequence alignments

Homology analyses were performed using the CLUSTAL multiple sequence alignment by MUSCLE (3.8) provided by EMBL-EBI [44].

## Results

### KoRVA and KoRVB envelope proteins show high homology to Gibbon Ape Leukemia Virus (GALV)

**Fig. 1:**
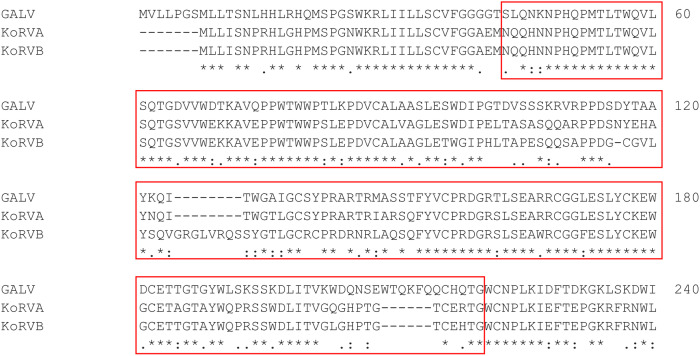
KoRVA and KoRVB sequences mainly differ from GALV in the RBD region. Multiple sequence alignment performed by MUSCLE, only amino acids 1–240 are depicted. GenBank identifiers: GALV: AAC96083.1; KoRVA: BAN63359.1; KoRVB: AGO86848.1. The RBD sequence is highlighted by the red boxes. Consensus amino acid sequence appears with asterisks below the alignment. Colons indicate conservation between groups of strongly similar properties - roughly equivalent to scoring >0.5 in the Gonnet PAM 250 matrix. Periods indicate conservation between groups of weakly similar properties - roughly equivalent to scoring ≤0.5 and >0 in the Gonnet PAM 250 matrix.

**Fig. 2:**
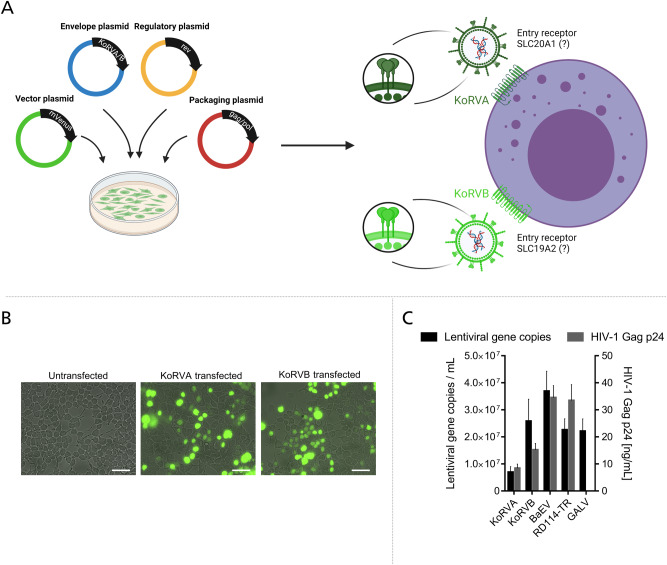
KoRVA and KoRVB pseudotyped lentiviruses can be successfully generated. **A** Schematic representation of lentivirus production using packaging plasmid gag/pol and regulatory plasmid rev, a transgene plasmid carrying the reporter gene mVenus as well as the envelope plasmid carrying KoRVA or KoRVB. On the right, representation of LV entry into an immune cell through alleged receptors SLC20A1 for KoRVA and SLC19A2 for KoRVB is depicted. **B** Representative images of HEK293T cells transfected with lentiviral plasmid mix. Green fluorescence depicts mVenus expression. Scale bar represents 50 µm. **C** Plot shows lentiviral gene copies per mL in lentiviral supernatant collected from HEK293T cells determined by qRT-PCR (black bars) and HIV-1 gag p24 protein (gray bars). Values from *n* = 6 independent experiments are shown.

As tools for an efficient and fast generation of ATMPs still remain elusive, we looked into the phylogeny of retro- and lentiviral envelope glycoproteins already in use in (pre-)clinical studies to identify novel viral envelope proteins which may potentially be used for genetic engineering of immune cells [45, 46]. In the phylogeny of gammaretroviral and lentiviral glycoproteins, we then examined two subtypes of KoRV due to their proximity to GALV which was employed as one of the first envelopes for producing pseudotyped viral particles [41]. Using multiple sequence alignment by MUSCLE, we identified a high homology within the receptor binding domain (RBD) sequence of the Koala retrovirus equivalents subtypes A and B to GALV (Fig. 1) [36, 47]. However, despite the close proximity on a phylogenetic level, the sequences of GALV, KoRVA and KoRVB deviate from each other, potentially impacting their efficacy to bind and infect immune cells, which prompted us to evaluate both subtypes. Out of 40 amino acids differing between KoRVA and KoRVB, 35 are located within the RBD sequence [40]. In consequence, KoRVA and KoRVB were reported to have different entry receptors, previously suggested to be PiT1/SLC20A1 and THTR1/SLC19A2, respectively (Fig. 2A). For GALV, PiT1/SLC20A1 was previously identified as an entry receptor, just like suggested for KoRVA [48].

### KoRV pseudotyped lentiviral vectors can efficiently be produced in HEK293T cells and transduce PBMCs

For the generation of pseudotyped lentiviral vectors, we used a third generation lentiviral system comprised of two transfer plasmids, gag/pol and rev, a transgene plasmid carrying a mVenus reporter gene and the envelope plasmid encoding either KoRVA or KoRVB. Cytopathic effects for producer cells of various pseudotyped lentiviral particles were described earlier [49]. Therefore, we assessed the HEK293T cells used for producing KoRVA and KoRVB pseudotyped LVs but could not find any indication for cytopathic effects (Fig. 2B). To check the formation of LVs, we assessed the content of p24 gag protein, which is incorporated as a structural protein into LV particles, via ELISA (Fig. 2C, gray bars). We observed p24 protein-specific signals from HEK293T supernatants after transfection with plasmids for the production of KoRVA and KoRVB pseudotyped LVs. Interestingly, with around 8.4 ng/mL, significantly less p24 gag protein was detected for KoRVA pseudotyped particles than for KoRVB, where we detected 15.2 ng/mL (*p* = 0.0022). BaEV and RD114-TR pseudotyped LVs showed significantly higher p24 gag protein content, with 34.5 ng/mL (*p* = 0.0238) and 33.5 ng/mL (*p* = 0.0043), respectively. As a control, we performed a single transfection of gag/pol plasmid into HEK293T cells which did not result in the release of substantial amounts of p24 gag protein into cell culture supernatants (0.4 ng/mL, not shown). To validate data from the p24 gag ELISA, LV particles were assessed in qRT-PCR to determine genome copy numbers of the viral particles. Of all envelopes, KoRVA pseudotyped LV showed the least amount of lentiviral gene copies, with an average of 6.955 × 10^6^ per mL. KoRVB LV showed 2.58 × 10^7^ gene copies per mL, BaEV 3.69 × 10^7^, RD114-TR 2.26 × 10^7^ and GALV 2.21 × 10^7^ (Fig. 2C). Gene copy numbers determined by qRT-PCR reflect protein data from ELISA well, suggesting that pseudotyping of LV with KoRVA produces fewer total virions per mL than pseudotyping with comparable envelope proteins such as KoRVB or BaEV and RD114-TR. According to our data, KoRVB is comparable to RD114-TR and GALV in terms of lentiviral gene copies, while BaEV pseudotyped virions yielded the highest titers.

**Fig. 3:**
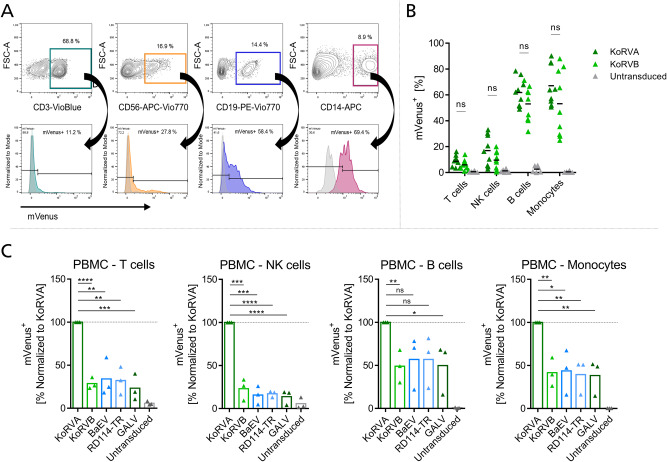
KoRVA and KoRVB pseudotyped lentivirus efficiently transduces various immune cell types in unstimulated PBMC. **A** Gating strategy for analysis of transduced PBMC. Upper panel: representative contour plots from one donor for gating of T cells, NK cells, B cells and macrophages. Lower panel: positively gated cells were then assessed for their reporter gene expression. Representative histograms showing mVenus expression within one cell type from one donor are depicted. Gray histograms represent untransduced controls. **B** mVenus expression was analyzed by flow cytometry on respective cell types found in PBMC transduced with mVenus encoding KoRV pseudotyped LVs; data from *n* = 9 independent experiments are shown. **C** mVenus+ cells were quantified in PBMC after transduction with LVs adjusted to 6.955 × 10^6^ gene copies per mL. Values are normalized to mVenus expression achieved by transduction with KoRVA LV. Values from *n* = 3 independently conducted experiments are depicted. *p ≤ 0.05, **p ≤ 0.01, ***p ≤ 0.001, ****p ≤ 0.0001.

We then used pseudotyped LVs to transduce freshly isolated PBMC to screen for immune cell types susceptible to KoRV. Three days after transduction, efficiency was analyzed by flow cytometry. The PBMC population was stained with antibodies specific for the four main cell types present: CD3 for T cells, CD19 for B cells, CD56 for NK cells and CD14 for macrophages (Fig. 3A). Upon transduction using KoRVA and KoRVB pseudotyped LVs on PBMCs, we observed an average efficiency of 17% versus 9.6% on NK cells, 61.9% versus 53% on B cells and 67.1% versus 53.2% on macrophages, using KoRVA and KoRVB, respectively. T cells were only transduced to 8.7% by KoRVA and 6% by KoRVB pseudotyped LVs (Fig. 3B). Next, we assessed the stability of KoRV pseudotyped LVs under two storage conditions to characterize prerequisites while working with KoRV. The stability of KoRV pseudotyped LVs was compared between storage at −80 °C to 4 °C after harvesting. In both cases, supernatants stored at 4 °C for one week did not show dramatic loss of performance compared to supernatants stored at −80 °C in terms of mVenus expression in the four subpopulations of PBMCs (Supplementary Fig. S1, 26.1% of primary NK cells vs. 29.9% for KoRVA and 12.1% vs. 13.3% for KoRVB; 5.7% of primary T cells vs. 8.9% for KoRVA and 3.7% vs. 4.4% for KoRVB; 53.2% of primary B cells vs. 50.5% for KoRVA; and 42% vs. 41.9% for KoRVB; 56.7% of primary monocytes vs. 59.7% for KoRVA and 30% vs. 20.9% for KoRVB). This remarkable stability of KoRV LVs may reduce the necessity of having large storage capacities at −80 °C when working with KoRV while simultaneously having advantages on observed efficiencies.

For direct comparison of KoRV to envelope proteins currently used in (pre)-clinical studies, LVs were adjusted to a gene copy number of 6.955 × 10^6^ per mL which relates to the gene copies measured for KoRVA LV. The positive cells for each population were then again gated for reporter gene expression. For evaluation, mVenus+ cells are visualized as percentages of cells efficiently transduced by KoRVA, which was set to 100% (Fig. 3C). Resulting from this analysis, T cells within PBMC were only transduced to 28.7% by KoRVB, 34.1% by BaEV, 32.2% by RD114-TR and 23.2% by GALV, in relation to KoRVA LVs, respectively. Average efficiencies of 23.1% by KoRVB, 15.7% by BaEV, 17.3% by RD114-TR and 13.8% by GALV on NK cells and 49.1% by KoRVB, 56.9% by BaEV, 56% by RD114-TR and 49.8% by GALV on B cells were achieved. For macrophages, 41.7% of mVenus+ cells observed with the use of KoRVA were reached by KoRVB, 43.6% by BaEV, 39.4% by RD114-TR and 38.3% by GALV, respectively. Our data suggest that especially within the NK cell fraction in PBMC, transduction with KoRVA pseudotyped LVs is up to 6-fold higher than with any of the other envelopes. On the other hand, freshly isolated monocytes and B cells are easier transduced than T cells and NK cells using the envelope proteins at hand which is indicated by higher values in comparison to KoRVA.

PBMCs were cultured in RPMI with 5% FCS without addition of cytokines, so no specific stimulation was performed. Following these first results generated in PBMCs, we decided to further look into transducing purified NK cells, B cells and monocytes with our novel KoRV LVs.

### KoRV pseudotyped LVs efficiently transduce freshly isolated Natural Killer cells

**Fig. 4:**
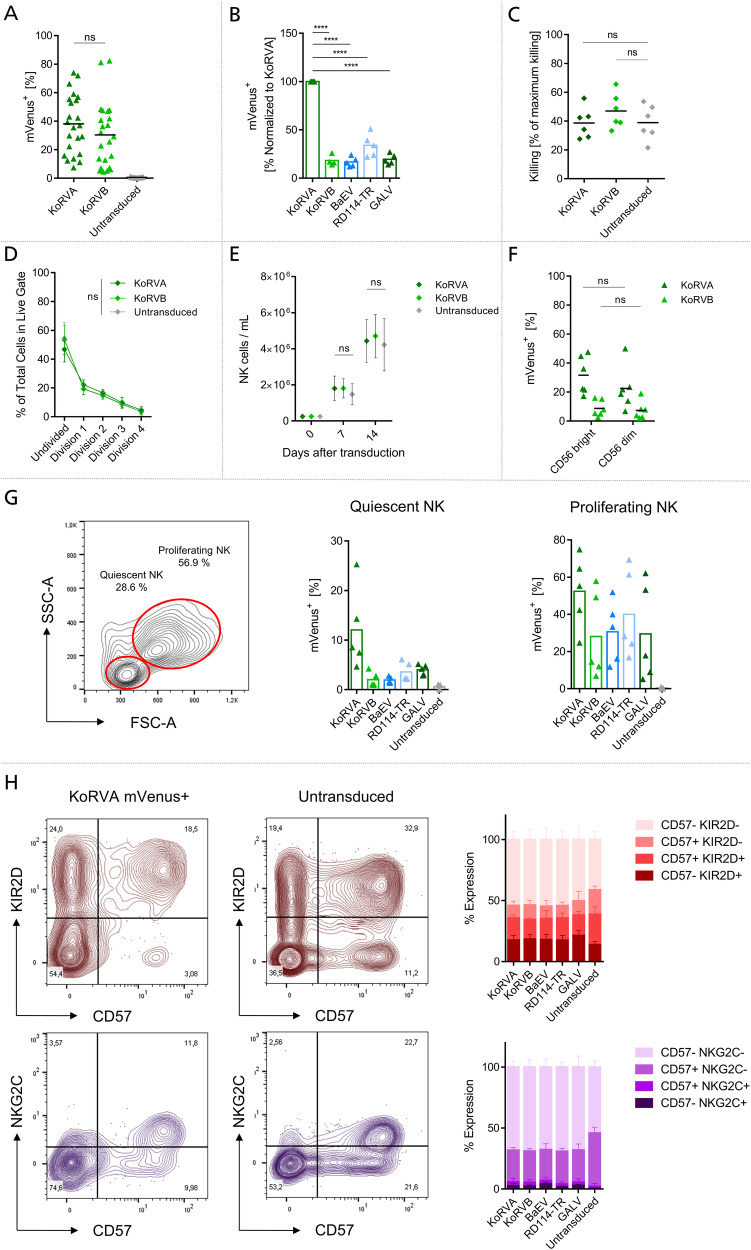
KoRVA and KoRVB pseudotyped LV efficiently transduce freshly isolated primary NK cells. **A** mVenus expression was analyzed by flow cytometry on freshly isolated NK cells transduced with mVenus encoding LVs three days after transduction. Values from *n* = 23 independently sampled donors are depicted. **B** mVenus+ cells were quantified after transduction with pseudotyped LVs adjusted to 6.955 × 10^6^ gene copies per mL. Values are normalized to mVenus expression achieved by transduction with KoRVA LV. Data from *n* = 5 independently conducted experiments are depicted. **C** NK cells were co-cultured with Calcein-labeled K562 cells. Lysis was assessed through the release of Calcein and is depicted as the percentage of maximum killing calculated through the measurement of spontaneous Calcein release as well as the maximum release initiated by Triton X-100. Individual values are shown as means from *n* = 6 independent experiments conducted in triplicates. **D** Proliferation of freshly isolated NK cells was monitored using CellTrace Violet staining and analyzed after four days of cultivation. The Flowjo proliferation tool was used to subdivide the peaks of NK cells subpopulations into cellular divisions and the percentage of the total cells counted on the MACSQuant X are depicted in the graph. Values are shown as mean with SD from *n* = 4 independent experiments. **E** Absolute quantification of NK cells proliferating after transduction for a period of 14 days. Data is represented as mean with SD from *n* = 5 independent experiments. **F** Expression of mVenus on CD56 bright and CD56 dim subsets of NK cells. Values from *n* = 6 independent experiments with two technical replicates are shown. **G** CD56+ NK cells were gated for single cell population by FSC-A vs FSC-H. Single cells were gated for proliferating and quiescent NK cells using FSC-A and SSC-A (left). mVenus+ cells were quantified in the subpopulations after transduction with LVs adjusted to 6.955 × 10^6^ gene copies per mL (right). Data from *n* = 5 independently conducted experiments are depicted. **H** Dot plots show representative receptor staining of maturation markers (CD57/KIR2D, top, red) and adaptive markers (CD57/NKG2C, bottom, purple) on a NKG2C+ NK cell donor (left, middle). mVenus+ NK cells transduced with KoRVA LV (left) and Untransduced cells (middle) are depicted. Receptor expression was quantified within subgates after transduction with various pseudotyped LVs (right). Data is represented as mean with SD from *n* = 4 independent experiments. *p ≤ 0.05, **p ≤ 0.01, ***p ≤ 0.001, ****p ≤ 0.0001.

In order to avoid competition for LV binding between the various immune cell types found in PBMC, we first isolated NK cells from PBMCs by negative selection and then transduced the freshly isolated cells with the same mVenus-encoding KoRV LV as in the previous experiment. In preliminary experiments, we compared the use of Vectofusin-1 and TransduceIT for enhancement of transduction and found Vectofusin-1 to yield higher efficiencies with KoRV pseudotyped LVs (Supplementary Fig. S2). KoRVA and KoRVB efficiently transduced freshly isolated NK cells with average efficiencies of 38.1% and 30.3% when applying undiluted LVs to the cells, respectively (Fig. 4A). Importantly, we observed a high donor variability using KoRV LVs. Still, both LVs applied in our experiment outperformed average transgene expression achieved by reported transduction using RD114-TR or BaEV LVs [37, 38]. Colamartino et al. reported an efficiency of 23% on freshly isolated NK cells for BaEV LV transduction with lower values for MV-LV and RD114-TR [37]. In our experiments, KoRVB reached an efficiency of 18.25%, while BaEV LV only reached an efficiency of 15% in direct comparison to KoRVA when adjusted to the same gene copy number per mL (Fig. 4B). In direct comparison, RD114-TR LV reached an efficiency of 32.8%, while GALV accomplished 17.8%. These trends are reflective of data generated in PBMC and further illustrate the supremacy of KoRVA over other envelope proteins tested in our setup.

To further study a potential activation of NK cells by KoRV pseudotyped LVs, we investigated additional phenotypic NK cell attributes. We co-incubated transduced NK cells with K562 cells which were labeled with the fluorescent, cell-permeant dye Calcein in a 1:1 ratio and monitored the release of Calcein from lysed K562 cells into the supernatant after two hours. Transduction of NK cells with either KoRVA or KoRVB did not result in significant changes in cytotoxicity towards target cells compared to untreated NK cells (Fig. 4C).

For tracking of proliferation, freshly isolated NK cells were stained with CellTrace Violet before transduction and proliferation was assessed in the following days (Fig. 4D). For neither KoRV treated condition, we observed differences to untransduced controls. Additionally, analysis of NK cell expansion over a course of 14 days after transduction revealed no differences between NK cells transduced with both KoRV pseudotyped LVs and control cells (Fig. 4E). KoRVA transduced NK cells reached a mean cell number of 4.43 × 10^6^, KoRVB transduced cells expanded to 4.7 × 10^6^ and untransduced cells reached 4.22 × 10^6^, resulting in a 17- to 19-fold expansion of NK cells using a feeder-free, cytokine-driven expansion protocol. Transduced mVenus+ NK cells were further characterized to determine the phenotype of NK cell populations susceptible for transduction by KoRV. Therefore, we analyzed the expression of the surface markers CD56 and CD16 and compared the ratios of CD56^bright^ and CD56^dim^ NK cell populations within transduced mVenus+ cells (Fig. 4F). NK cells in both subsets did not show significant differences in mVenus expression with 31.6% mVenus+ and 22.4% mVenus+ in KoRVA LV and 8.7% and 7.3% in KoRVB LV transduced cells.

In contrast, we identified diverging potential regarding transducibility between the proliferating subset of NK cells characterized by higher granularity and larger size and the quiescent population defined by smaller size (Fig. 4G). While quiescent CD56+ NK cell populations transduced with gene copy number adjusted LVs show only poor mVenus expression (12.1% KoRVA, 2.0% KoRVB, 1.9% BaEV, 3.6% RD114-TR, 4.0% GALV), proliferating and larger CD56 + NK cells show mVenus expression of 52.4% for KoRVA LV, 28.1% for KoRVB, 30.6% for BaEV, 40.0% for RD114-TR and 29.5% for GALV.

Next, NK cells were characterized for the expression of maturation markers as this is one means to define adaptive NK cells [50]. Proliferative NK cells, as defined above, generally showed lower expression of maturation markers KIR2D, CD57 and NKG2C when compared to quiescent cells. Consequently, transduction efficiency in KIR2D^hi^, CD57^hi^ and NKG2C^hi^ cells was lower than in cells with low receptor expression. Individually, KIR2D and CD57 expression was always considerably lower across 5 donors for transduced mVenus+ NK cells compared to untransduced in all pseudotypes (Fig. 4H). For all pseudotyped LVs, double negative populations ranged from 50.5 to 54.7% for CD57/KIR2D in Transduced mVenus+ cells vs 41.4% in untransduced and from 67.9% to 69.3% vs 54.2% in untransduced for CD57/NKG2C, respectively. Only one of the donors which was investigated showed NKG2C^hi^ expression (Fig. 4H, Supplementary Fig. S3). However, in line with observations made for KIR2D and CD57, NKG2C expression was also lower in transduced mVenus+ cells in this donor than in untransduced cells. Hence, our data show that NK cells which were the most susceptible to KoRV also showed low expression of KIR2D, CD57 and NKG2C as well as higher proliferation rates defined by cell size.

Expression of further receptors was assessed on untreated cells (Untransduced) as well as KoRV treated cells which remained either untransduced (Transduced mVenus−) despite the application of KoRV pseudotyped LVs or were efficiently transduced with measurable reporter gene expression (Transduced mVenus+). Differential expression could be observed for NKG2A, NKp30 and CD69 for both KoRVA and KoRVB and NKG2D only for KoRVB (Supplementary Fig. S4). Differences were predominantly observed comparing untreated and mVenus+ cells, attributing part of the altered expression to the general lentiviral transduction of NK cells. However, smaller differences were also observed between mVenus− and mVenus+ cells. These changes could possibly be attributed to different subsets of NK cells, which are either more proliferative or more quiescent, as it was also already observed earlier by Bari et al. [38]. The higher expression of CD69 as an activation marker also hints at a more activated state of mVenus+ cells. However, expression of additional activation markers like NKp44 did not differ between groups.

### KoRV pseudotyped LVs efficiently transduce primary monocytes

**Fig. 5:**
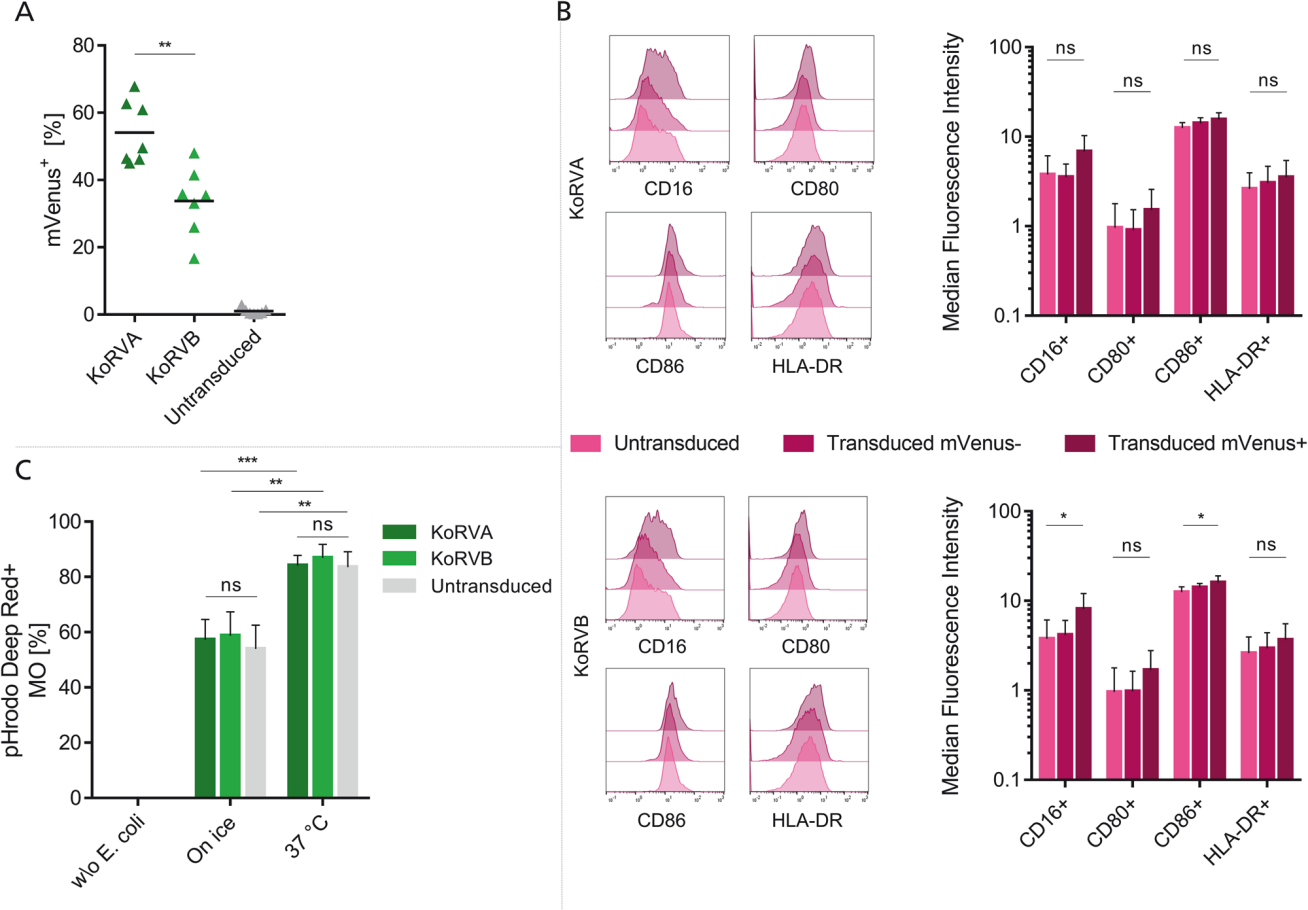
KoRVA and KoRVB pseudotyped LV efficiently transduce freshly isolated primary monocytes. **A** mVenus expression was analyzed by flow cytometry on freshly isolated monocytes transduced with KoRVA and KoRVB pseudotyped mVenus encoding LVs three days after transduction. Values from *n* = 7 independently conducted experiments are depicted. **B** Representative histograms of one donor show receptor staining in untreated cells, mVenus− and mVenus+ cells. Bar plots show means with SD of Median Fluorescence Intensity of each receptor staining of *n* = 6 donors. **C** Transduced monocyte-derived macrophages were incubated with pHrodo Deep Red labeled E. coli for one hour either at 4 °C or 37 °C in an incubator and fluorescence was measured on the macrophages using a flow cytometer, indicating how many macrophages phagocytosed E. coli. *p ≤ 0.05, **p ≤ 0.01, ***p ≤ 0.001, ****p ≤ 0.0001.

After transducing NK cells, we next looked at the potential of KoRV pseudotyped LVs to transduce freshly isolated monocytes. Therapies involving macrophages hold lots of potential for the treatment of various diseases and present an emerging field of research [16]. As the impact of KoRVA and KoRVB gene copy numbers were already assessed on monocytes in PBMC, we limited ourselves to the description of KoRV in this section. Transduction of freshly isolated monocytes using KoRVA led to 54.1% mVenus transgene expression while KoRVB yielded 33.8% of mVenus+ cells (Fig. 5A). Therefore, KoRVA has a significantly higher potential for transducing freshly isolated monocytes (*p* = 0.0022).

Cultivation of monocytes in the presence of granulocyte macrophage colony-stimulating factor (GM-CSF) results in the differentiation into proinflammatory, M1-like macrophages [51]. These macrophages show a more proinflammatory phenotype compared to macrophages stimulated with M-CSF which act anti-inflammatory [52, 53]. To investigate whether KoRV pseudotyped LVs have undesired effects on the resulting macrophages after differentiation and activation of monocytes after three days of culturing, several surface markers were evaluated post-transduction (Fig. 5B). We observed a slightly higher expression of CD16 and CD86 following transduction between untreated and transduced mVenus+ cells, which contribute to the activation of antibody-dependent cellular cytotoxicity (ADCC) and of activation of bystander lymphocytes, respectively [54, 55].

Next, to assess the innate functionality of transduced macrophages, a phagocytosis assay was conducted. pHrodo Deep Red labeled E. coli were incubated with macrophages for 60 min either on ice to determine unspecific binding of E. coli to macrophages as well as background phagocytosis or at 37 °C to record the level of active phagocytosis. Macrophages without E. coli served as a negative control (Fig. 5C). In our experiments, we observed a background signal of phagocytosis of 57.3% and 58.7% for KoRVA and KoRVB, respectively, with untransduced macrophages showing 54% of phagocytosis. After incubation at 37 °C for one hour, more than 80% of macrophages appeared Deep Red positive in the flow cytometer which represents a significant increase for both KoRV conditions as well as the untransduced control (84.1% for KoRVA, 86.9% for KoRVB and 83.5% for untransduced control). We also assessed the amount of engulfed bacteria by assessing the median fluorescence intensity. Interestingly, we observed the highest uptake rates for KoRVB modified macrophages in 3 out of 4 donors (Supplementary Fig. S5), which may be in line with the slightly elevated activation status observed in the flow cytometry phenotyping. Taken together, lentiviral transduction of macrophages did not alter their phenotype or impair their innate function in phagocytosis.

### KoRV pseudotyped LVs efficiently transduce freshly isolated B lymphocytes

B cells have been shown to play both anti- and pro-tumorigenic roles in tumor tissues. B lymphocytes are involved in the regulation of T cells and innate immune responses and therefore hold the potential to add new perspectives to immunotherapies. Tumor-resident B cells can produce antibodies against self-antigens overexpressed in cancer [56]. Additionally, there are attempts to engineer B cells, e.g. to secrete pathogen-specific antibodies [57] or immune regulatory molecules [58].

**Fig. 6:**
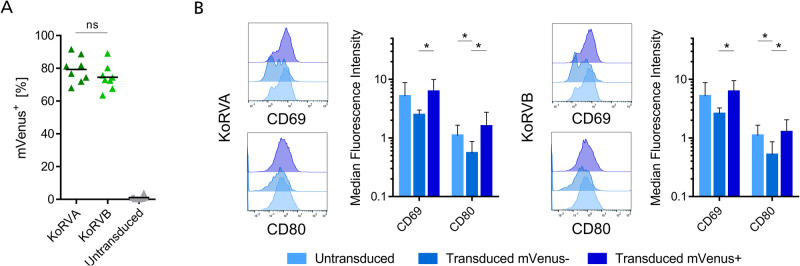
KoRVA and KoRVB pseudotyped LV efficiently transduce freshly isolated primary B cells. **A** mVenus expression was analyzed by flow cytometry on freshly isolated B cells transduced with mVenus encoding LVs three days after transduction. Values from *n* = 8 independently conducted experiments are depicted. **B** Representative histograms of one donor show receptor staining in untreated cells, mVenus- and mVenus+ cells. Bar plots show means with SD of Median Fluorescence Intensity of each receptor staining of *n* = 7 donors. *p ≤ 0.05, **p ≤ 0.01, ***p ≤ 0.001, ****p ≤ 0.0001.

Freshly isolated B cells were transduced using KoRVA and KoRVB pseudotyped LVs and yielded efficiencies of 79.3% and 74.5%, respectively (Fig. 6A). The highest measured transduction efficiency in our experiments even reached more than 90% of mVenus expressing cells following transduction.

To investigate the activation state of transduced B lymphocytes three days after transduction, we looked at CD69 and CD80 expression. Here, untreated and mVenus+ B cells have very comparable median values for both surface receptors, however mVenus- B cells have significantly lower median values than mVenus+ cells and also in part compared to untreated B cells (Fig. 6B).

## Discussion

Lots of viral envelope proteins used in biotechnology bind membrane-transporters to infiltrate human cells. ASCT1 is used by RD114-TR while BaEV uses ASCT2 in addition to ASCT1 as entry receptors [59]. The sodium-dependent phosphate transporter 1 (PiT1 or SLC20A1) has been described as an entry receptor for both GALV and KoRVA, while KoRVB employs the thiamine transporter 1 (THTR1 or SLC19A2) for infection of host cells [40], which sparked an interest in regard to their potential to enable viral entry into immune cells. VSVG uses the low-density lipoprotein receptor (LDL-R) as entry receptor [60], which is not or lowly expressed on resting immune cells [61]. The expression patterns of various receptors on host cells enable viruses to infect cells at specific stages of activation and proliferation. However, despite presumably sharing the same entry receptor, the apparent differences in the tropism of KoRVA and GALV and their ability to infect human immune cells which we demonstrate in this study, argue that PiT1 alone is not sufficient to mediate cell entry. We speculate that either a different entry receptor exists, or additional, previously undescribed factors other than the envelope-receptor interaction strongly influence the infectivity of LVs in these cells.

In qPCR experiments conducted with cDNA generated from freshly isolated cell populations of T cells, NK cells, B cells and monocytes, we could not find any correlation of the SLC20A1 receptor mRNA levels to the capability of KoRVA and KoRVB to efficiently transduce specific cell types (data not shown). According to our data, NK cells had more mRNA copies of the receptor, while the overall transduction efficiencies were higher for both B cells and monocytes at this time. Our own data is in line with SLC20A1 levels deposited in the DICE RNA expression database (as of 2024), showing highest levels in NK cells, followed by T cells and macrophages, while B cells have very low levels of SLC20A1 RNA [62]. Additionally, we performed an overexpression of PiT1 on HEK293T and K562 cells, however could not confirm that the overexpression led to increased reporter gene expression when transducing with KoRVA pseudotyped particles (data not shown), in line with a study from 2006 which showed PiT1-dependence of GALV, but not KoRV [63]. This again argues that either PiT1 by itself may be insufficient to ensure successful KoRVA entry and/or additional cellular factors are crucial for successful cell infiltration.

Noteworthy, multiple versions of the KoRVA and KoRVB envelope proteins have been described until now. For the creation of our envelope plasmids we used sequences provided by Xu et al. [40, 64]. Today, further KoRV subtypes ranging until KoRVI have been described which partly differ in their assigned entry receptors or entry receptors have not been described yet [64]. Exploring further subtypes of KoRV in the future might reveal even more potent envelope versions for the transduction of freshly isolated immune cell types.

The ex vivo manufacturing of all currently approved CAR-T cell therapy products as well as current protocols to generate CAR NK cells rely on the use of retroviral particles after immune cell activation [46, 65]. Shortening and streamlining processes for cell manufacturing significantly decreases costs and frees up limited manufacturing capacities [66].

The transduction of freshly isolated monocytes, NK cells and B cells right after isolation holds several advantages over the transduction of pre-activated immune cells: (i) Simpler manufacturing strategies with minimized process steps as cells don’t have to be collected for the transduction (ii) Virus amounts required are minimized as cells are transduced in lower numbers prior to expansion.

Additionally, KoRV LVs can be produced with standard protocols and reagents for lentivirus manufacturing, and have a profound stability, as we observed stable transduction rates with lentiviral supernatants stored for one week at 4 °C compared to the same viral stock immediately frozen and stored at −80 °C (Supplementary Fig. S1). This can be explained by the damaging effect of the freezing process on the virus particles which was reported before for other vectors, however, despite the known titer loss after freezing, this storage option is highly recommended for LVs due to their relatively low stability at 4 °C [67, 68]. The option to store KoRV at 4 °C may further facilitate the production of allogeneic cell products.

Compared to other envelopes, KoRVA pseudotyped LVs generated low viral titers as judged by low HIV-1 p24 gag protein content and lentiviral genome copy numbers. To improve titers in future applications, the envelope protein could be codon-optimized [69]. In this study, we decided to use the wild-type sequences of KoRVA and KoRVB, as codon-optimization was previously shown to have deleterious effects on the final titers for some other viral envelopes due to alterations in mRNA structure and stability or improper glycosylation [70]. Furthermore, ratios between packaging, transgene and envelope plasmids are crucial for obtaining high titers and might influence the number of virions produced when using KoRV for pseudotyping, however they were kept at the same ratio for all envelopes to ensure comparability between LVs [71, 72]. We did not perform experiments to exactly determine the ratio between viable to non-viable particles.

An additional advantage of KoRV is the absence of obvious cytopathic effects in HEK293T producer cells. Both in VSVG producing HEK293T cells as well as BaEV producing cells, cytopathic effects like syncytium formation and detachment of HEK293T producer cells were described [73, 74]. Whereas cytopathic effects are not very critical for lab-scale production of LVs using transient transfection, for cell therapy manufacturing, usually, stable producer cells are being generated which continuously secrete viral particles in defined amounts and quality. For VSVG, huge efforts were taken to overcome cytopathic effects and enable the manufacturing of clinical scale LVs [75–77]. For KoRV LVs, the process to generate stable expressers and scale up production should be much simpler. All the above-mentioned advantages facilitate the production of LVs without compromising on the quality of the final cell therapy product.

None of our experiments revealed a significant impact of KoRV LV transduction on the biological properties of NK cells, B cells or monocyte-derived macrophages. Phenotypically, we observed in all cell types the trend that reporter-positive cells showed a slight increase in activation markers. However, we cannot distinguish if cells are being activated in response to viral infection, or if certain immune cells with a pre-activated phenotype in blood are preferentially transduced. Of note, while transduction efficiencies for monocytes and B cells were fairly uniform between different PBMC donors, NK cell transductions showed huge donor dependency.

It was recently shown for cytokine-expanded NK cells that BaEV predominantly infects highly proliferative, less mature cells [38, 78], which is in line with observations we made concerning the ability of KoRVA and KoRVB to mainly transduce proliferative NK cells with low expression of KIR2D, CD57 and NKG2C [79, 80]. This certainly adds to the predisposition defining the potential of donors for efficient transduction. Other than that we could not resolve additional patterns of surface markers predetermining the transduction efficiency prior to transduction. It is possible that donor variation in fresh NK cells may even surpass variations of uniformly-activated, cytokine-exposed NK cells in culture. Fresh NK cells may show higher phenotypic variation, e.g. based on recent or chronic infections that shape the NK cell repertoire or ratios between quiescent and proliferating cells [81]. Therefore, it may well be that for NK cell based allogeneic cell products, several donors have to be tested for their suitability as donors not only in terms of cellular functionality, but also for expansion rates and transduction rates of NK cells.

Next to NK cells, also macrophages pose a promising cell type currently evaluated for cancer therapy. Previously, CAR macrophages have been generated using adenoviruses, which resulted in a M1-polarized phenotype, tending towards more proinflammatory reactions [24]. However, adenoviral plasmids are cumbersome to work with and few laboratories hold the respective know-how, whereas LV generation is a commonly used standard method. Through the use of KoRVA and KoRVB for lentiviral transduction on the day of monocyte isolation, the possibility to engineer monocytes even before a final differentiation is possible. Both M1 and M2 polarized macrophages have distinct features that benefit different treatment options, so influencing the polarization before transduction poses great opportunities [82, 83].

KoRV pseudotyped LVs enabled also the modification of B cells already early on after the isolation process without prior activation. These high transduction rates of around 80% have not been described before to our knowledge. Levy et al. reported high transduction efficiency using BaEV-LVs only upon B cell receptor stimulation which was not necessary in our approach as we transduced the cells right after isolation [84]. In resting B cells, only around 40% of B cells were efficiently transduced. A different study compared VSVG and measles envelope pseudotyped LV on quiescent B cells and also reached around 40% transgene positive cells for the superior measles envelope [85]. This may enable the generation of genetically engineered naive B cells that may in vivo differentiate upon antigen exposure. Due to the exceptional longevity of plasma B cells which can reside within the bone marrow for decades, and their ability to secrete substantial protein levels, these cells could also be harnessed as an engineered cell therapy for protein replacement [86]. This innovative cell therapy might outperform current protein delivery methods that necessitate frequent dosing. Protein medications are widely employed to treat various conditions, including cancer, autoimmune disorders and protein deficiencies [86, 87]. Additionally, efficient transduction using KoRV LV represents a handy basic research tool in terms of gene overexpression in resting primary cells which makes the use of KoRV interesting in a broad range of applications [88].

In summary, we showed that KoRVA and KoRVB pseudotyped LVs are efficient tools for the transduction of freshly isolated human NK cells, B cells and monocytes and therefore hold the potential to advance manufacturing processes for a quick, simple and cost-efficient generation of allogeneic cell therapy products. Additionally, these LVs can be used as a research tool to accelerate the development of innovative allogeneic cell therapies beyond T cell engineering and may take allogeneic cell therapies to the next level.

### Supplementary information

Supplementary Information File

## Data Availability

All relevant data are included within the published article and its supplementary files. Raw data are available on request from the corresponding author.
