# Supplementary material for: Development of KoRV-pseudotyped lentiviral vectors for efficient gene transfer into freshly isolated immune cells
Source: Gene Ther. 2024 Apr 29;31(7-8):378–90. doi: 10.1038/s41434-024-00454-0 (PMC11257948; doi:10.1038/s41434-024-00454-0)
Supplement: Supplementary file 1 — Supplementary Information File [file 41434_2024_454_MOESM1_ESM.pdf]

SUPPLEMENTARY DATA

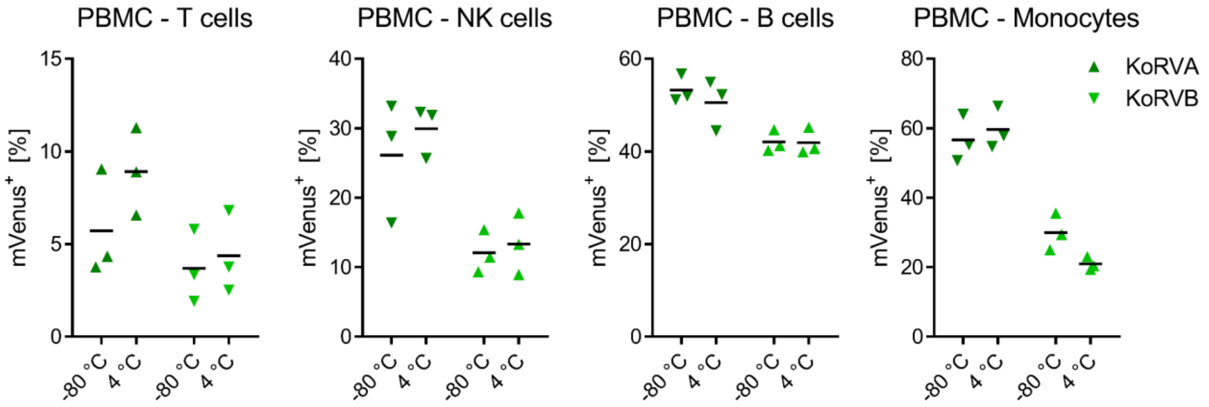

Figure S1: Comparison of pseudotyped LVs stored at -80 °C versus 4 °C after harvesting from HEK293T producer cells. Freshly isolated PBMC were used for transduction. Values from n = 3 independent experiments with two technical replicates are shown.

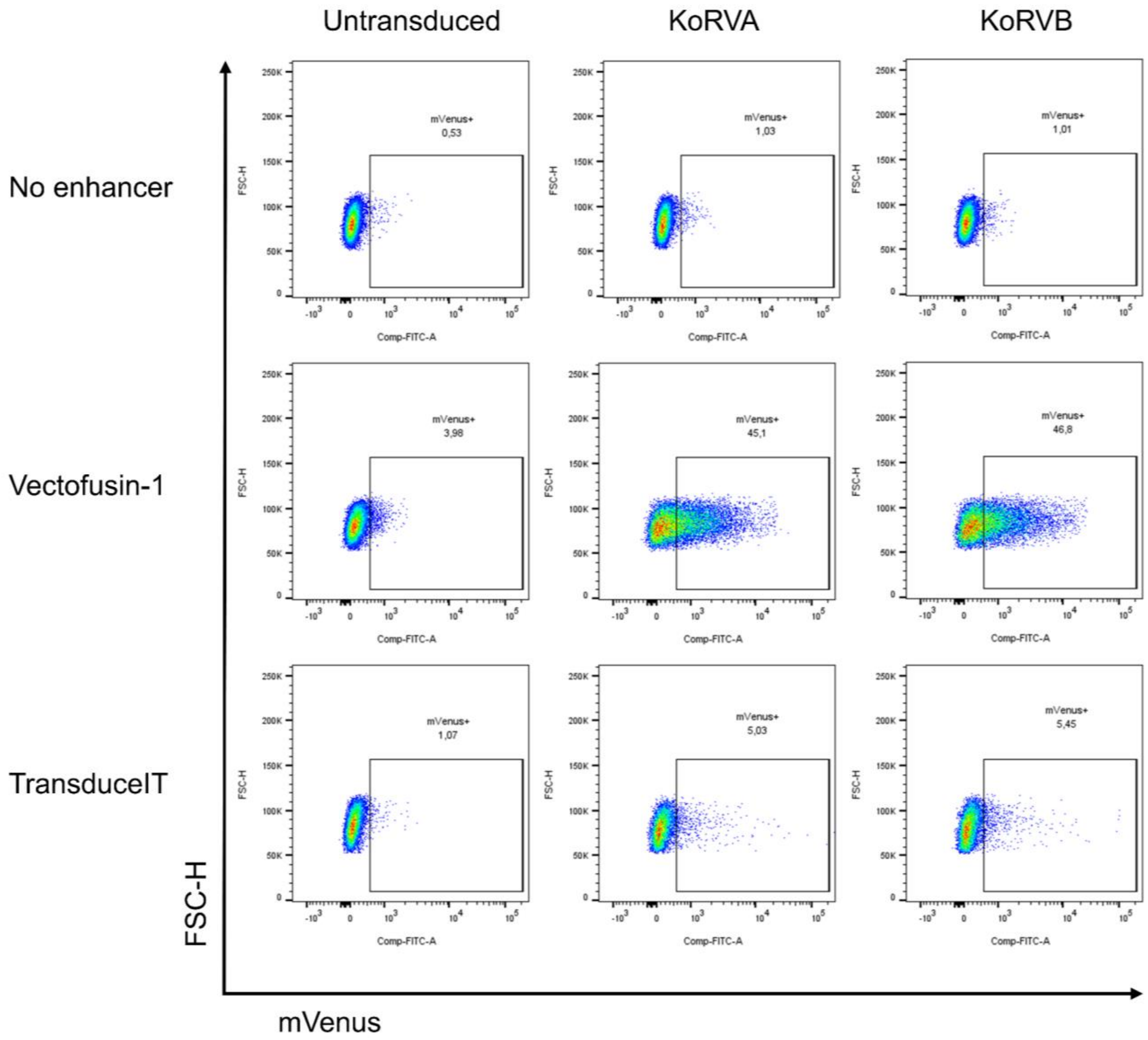

Figure S2: Gating strategy for detection of reporter gene positive NK cells. Freshly isolated NK cells were isolated from buffy coats and transduced with KoRV pseudotyped LVs and the addition of either Vectofusin-1 or TransduceIT as transduction enhancers; mVenus expression was analyzed by flow cytometry three days after transduction and is depicted on the x-axis.

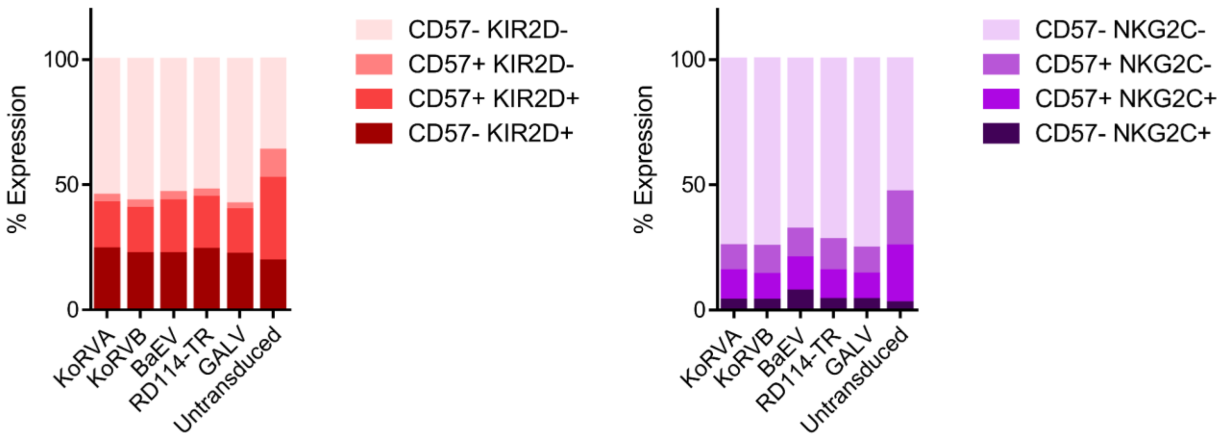

Figure S3: mVenus+ NK cells transduced with pseudotyped LVs are depicted. Receptor expression for maturation markers (CD57/KIR2D, top, red) and adaptive markers (CD57/NKG2C, bottom, purple) on a NKG2C+ NK cell donor was quantified within subgates after transduction with pseudotyped LVs.

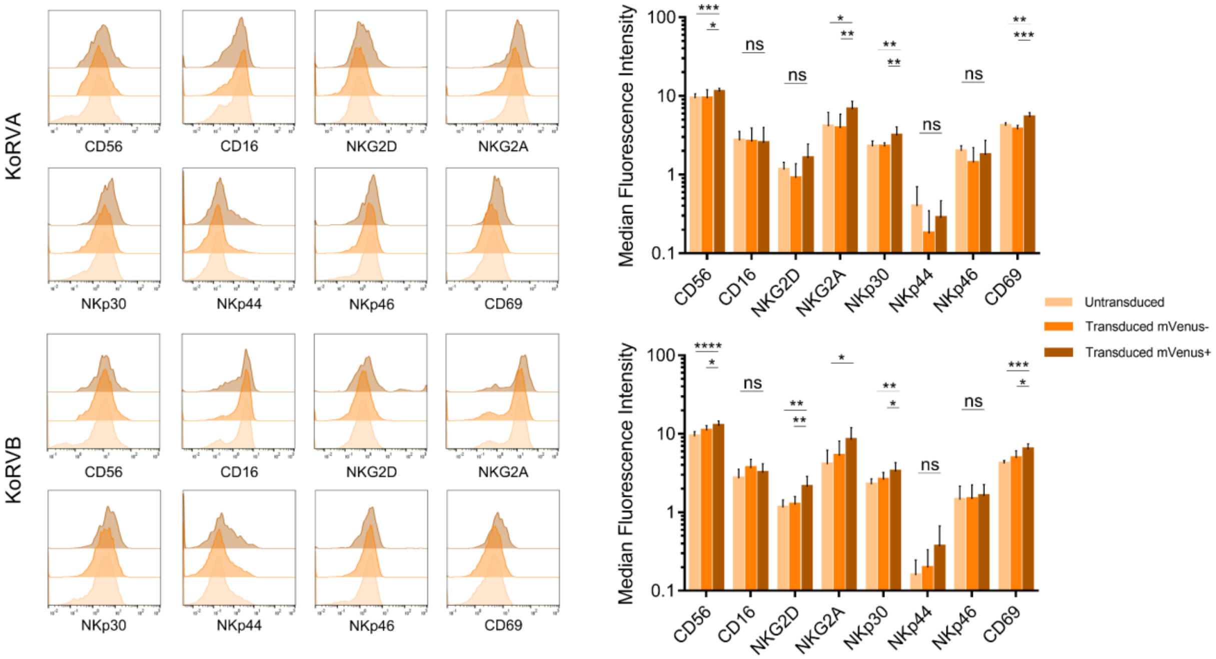

Figure S4: NK cell receptor expression was assessed following transduction. Histograms show representative receptor staining in untreated cells (Untransduced), mVenus- (Transduced mVenus-) and mVenus+ (Transduced mVenus+) cells. Bar plots show Median Fluorescence Intensity of each receptor. Mean values with SD are shown from n = 6 independent experiments. * = $p \leq 0.05$, ** = $p \leq 0.01$, *** = $p \leq 0.001$, **** = $p \leq 0.0001$.

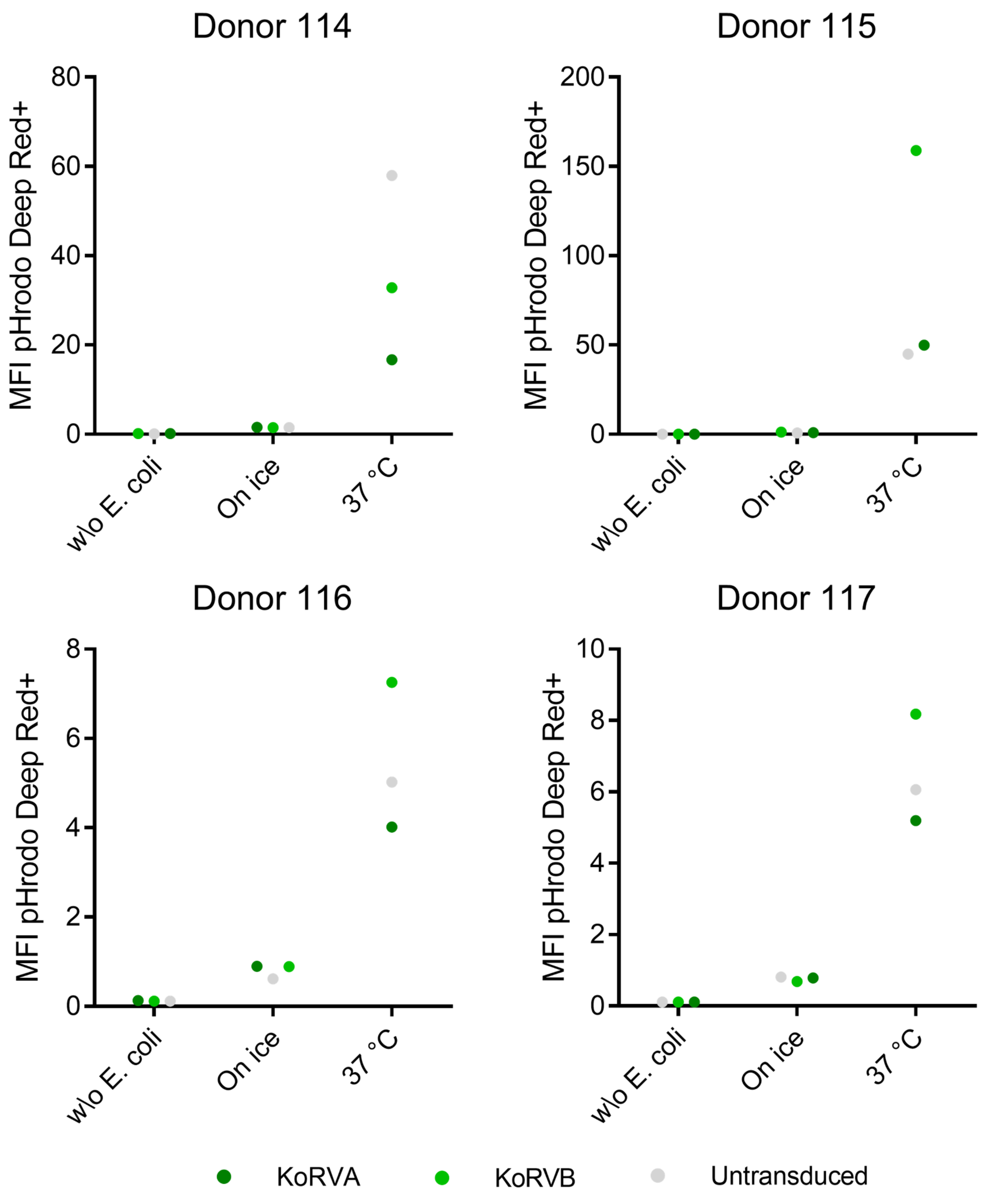

Figure S5: Transduced macrophages were incubated with pHrodo Deep Red labeled E. coli for one hour either at 4 °C or 37 °C in an incubator and Median Fluorescence Intensity was measured on the macrophages using a flow cytometer, indicating the amount of phagocytosed E. coli.
